# Subatomic resolution X-ray structures of green fluorescent protein

**DOI:** 10.1107/S205225251900246X

**Published:** 2019-04-03

**Authors:** Kiyofumi Takaba, Yang Tai, Haruhiko Eki, Hoang-Anh Dao, Yuya Hanazono, Kazuya Hasegawa, Kunio Miki, Kazuki Takeda

**Affiliations:** a Department of Chemistry, Graduate School of Science, Kyoto University, Sakyo-ku, Kyoto 606-8502, Japan; b Protein Crystal Analysis Division, Japan Synchrotron Radiation Research Institute (JASRI), 1-1-1 Kouto, Sayo-cho, Sayo-gun, Hyogo 679-5198, Japan

**Keywords:** protein structure, refinement, X-ray crystallography, hydrogen bonding, green fluorescent protein

## Abstract

Green fluorescent protein (GFP) is a light-emitting protein that does not require a prosthetic group for its fluorescent activity. As such, GFP has become indispensable as a molecular tool in molecular biology. Nonetheless, there has been no subatomic elucidation of the GFP structure owing to the structural polymorphism around the chromophore. Here, subatomic resolution X-ray structures of GFP without the structural polymorphism are reported. The positions of H atoms, hydrogen-bonding network patterns and accurate geometric parameters were determined for the two protonated forms. Compared with previously determined crystal structures and theoretically optimized structures, the anionic chromophores of the structures represent the authentic resonance state of GFP. In addition, charge-density analysis based on atoms-in-molecules theory and noncovalent interaction analysis highlight weak but substantial interactions between the chromophore and the protein environment. Considered with the derived chemical indicators, the lone pair–π interactions between the chromophore and Thr62 should play a sufficient role in maintaining the electronic state of the chromophore. These results not only reveal the fine structural features that are critical to understanding the properties of GFP, but also highlight the limitations of current quantum-chemical calculations.

## Abstract 2

Subatomic resolution X-ray structures of green fluorescent protein are reported.

## 1. Introduction   

**Figure 1:**
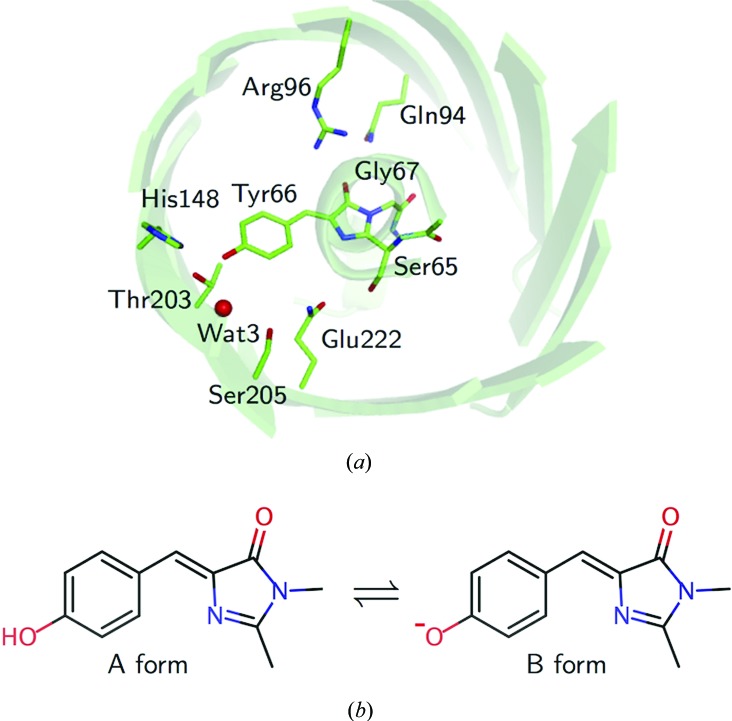
The structure of GFP from *A. victoria*. (*a*) The GFP structure as viewed from the top of the β-barrel, showing the chromophore and the interacting residues. The residue names are shown for wild-type GFP, while the structure of the E222Q variant determined in this research is used as the model. (*b*) The dual protonation states of the chromophore.

Green fluorescent protein (GFP), which was discovered in the jellyfish *Aequorea victoria*, is a light-emitting protein consisting of 238 amino acids. GFP absorbs UV–blue light and emits green light. The structure consists of an 11-stranded β-barrel plugged by a chromophore [Fig. 1 ▸(*a*)]. The chromophore is 4-(*p*-hydroxybenzylidene)imidazolin-5-one, which is formed from three intrinsic residues (Ser65, Tyr66 and Gly67) in the polypeptide chain by a post-translational cyclization, dehydration and oxidation. There are dual protonation states of the hydroxybenzyl (tyrosyl) group of the chromophore in the protein. The protonated neutral ‘A’ and deprotonated anionic ‘B’ forms [Fig. 1 ▸(*b*)] are present at ratios of 6:1 to 4:1 in wild-type GFP (Brejc *et al.*, 1997 ▸; Chattoraj *et al.*, 1996 ▸). The two forms have distinct spectroscopic properties (Chattoraj *et al.*, 1996 ▸). The absorption peak at 398 nm belongs to the A-form chromophore, while the peak at 475 nm belongs to the B form [Supplementary Fig. S1(*a*)].

Wild-type GFP and its homologs, which are collectively called fluorescent proteins (FPs), have become indispensable in current molecular biology as tools for monitoring gene expression and protein localization (Chudakov *et al.*, 2010 ▸). Moreover, FPs are thought to be candidates for use in quantum devices (Shi *et al.*, 2016 ▸; Dietrich *et al.*, 2016 ▸). In each of these cases, researchers have used random mutagenesis to customize FPs and extend their functions. A folding variant, cycle3 GFP, is representative (Crameri *et al.*, 1996 ▸). Researchers have also tried to design novel FP variants to meet their requirements, coupled with the development of computational simulations (Amat & Nifosì, 2013 ▸). Such theoretical approaches are especially important in changing the spectroscopic features of FPs, because the effects of amino-acid substitutions on the electronic state of the chromophore must be considered. Because the electronic structure of the chromophore is strongly related to its interactions with the protein environment, accurate structural information is indispensable if functional modifications are to be successful. Since the first crystal structure of an FP was reported at 1.9 Å resolution (Ormö *et al.*, 1996 ▸), structural studies on FPs have continuously been performed and their resolutions have been updated. In the earlier studies (Brejc *et al.*, 1997 ▸; Palm *et al.*, 1997 ▸), it was proposed that the dual protonation states of the chromophores are coupled to their hydrogen bonding to surrounding residues. The role of each residue in regulation of the hydrogen-bonding network has been verified by mutagenesis, particularly of Ser65, His148, Thr203 and Glu222 (Cubitt *et al.*, 1995 ▸; Shu *et al.*, 2007 ▸; Kummer *et al.*, 2000 ▸; Elsliger *et al.*, 1999 ▸). The highest-resolution structure of 0.9 Å was achieved for a variant of GFP (Shinobu *et al.*, 2010 ▸). This suggested that protons may have been supplied from the surface to the chromophore (Agmon, 2005 ▸). In the structure, H atoms were indeed modeled. However, the protonation of dissociable groups was based not on the electron density but on a computational prediction. As a result, while the A and B forms have been reported to exist in a nearly 1:1 ratio in solution, the tyrosyl group of the chromophore was modeled to be protonated in spite of the anionic features of the geometry of the non-H atoms. It was attempted to resolve this situation by using molecular-dynamics simulations (Shinobu & Agmon, 2017 ▸). Nonetheless, the existence of H atoms at the crucial sites and the relationships between the donors and acceptors of hydrogen bonds have remained ambiguous in the absence of experimental data. Moreover, a high-resolution crystal structure has not been reported for a GFP variant with only a neutral chromophore in its absorption spectrum. Therefore, highly reliable geometric parameters for the two forms of FPs have not been made available, although such parameters would be a milestone in understanding the mechanism behind their spectroscopic properties. There have been a number of computational studies of FPs that have elucidated specific interactions affecting the electronic state of the chromophore. Together, these studies have allowed us to approach this topic despite the dearth of experimental data. Meanwhile, these computational findings should be verified by comparing them with a highly accurate determination of the experimental geometry, since the theoretical approach generally includes geometric optimization.

When determining the structure of FPs using crystallo­graphy, we should give special consideration to the effect of radiation. Adam and coworkers showed that X-ray irradiation induces a loss of planarity in the chromophore and simultaneous bleaching of IrisFP, a photoactivatable homolog (Adam *et al.*, 2009 ▸). For F64L/S65T, a photostable variant of GFP, two apparent structural alterations were reported: the decarboxyl­ation of Glu222 and the deviation of coordinated waters near the chromophore (Royant & Noirclerc-Savoye, 2011 ▸). In these studies, such structural disturbances were observed at X-ray doses of ∼5 × 10^5^ Gy at 100 K, which are below the Henderson limit of 2 × 10^7^ Gy (Henderson, 1990 ▸). The chromophore and the surrounding residues and waters are extremely sensitive to radiation (Fioravanti *et al.*, 2007 ▸). Hence, it is possible to consider an authentic FP structure that not only has accurate coordinates but also sufficiently avoids the effect of radiation.

In order to elucidate the subatomic features of the chromophore in the protein environment, we performed ultrahigh-resolution X-ray crystallographic analyses of both protonation states. For the study of the A form, we used a T203I variant (Cubitt *et al.*, 1995 ▸) in which the side chain of Ile203 mimics the conformation of Thr203 in the A form of wild-type GFP (Brejc *et al.*, 1997 ▸). We used two different variants, S65T and E222Q, for the B form. The S65T substitution changes the conformation of Glu222 by relocating the hydroxyl group of the 65th residue. The donors and acceptors in the hydrogen bonds among Glu222, Ser205, a water and the chromophore switch with each other from those in the A form of wild-type GFP. Consequently, the chromophore becomes anionic, while Glu222 becomes neutral (Brejc *et al.*, 1997 ▸). On the other hand, the E222Q variant maintains the chromophore in the anionic state by replacing Glu222 with a neutral glutamine. This variant is useful for investigating the features of the B form (Elsliger *et al.*, 1999 ▸; Abbruzzetti *et al.*, 2005 ▸), although the maturation of its chromophore is significantly dependent on pH (Sniegowski *et al.*, 2005 ▸). The hydrogen-bond network between Tyr66 and Glu222 is thought to be disrupted in the E222Q variant as well as in the S65T variant (Stoner-Ma *et al.*, 2008 ▸). Thus, we complementarily used these two variants to analyze the features of the B form.

Interactions in protein molecules are generally estimated only from the geometric information, such as bond lengths and angles. However, by using the electron density at an ultrahigh resolution of better than ∼0.80 Å and charge-density analysis based on the atoms-in-molecule (AIM) theory (Bader, 1990 ▸), intramolecular interactions can be detected directly as features of the electron density. Although charge-density analysis has traditionally been used to quantify the intermolecular interactions in the crystals of small compounds (Koritsanszky & Coppens, 2001 ▸), a few studies have applied charge-density analysis to protein molecules (Hirano *et al.*, 2016 ▸; Takaba *et al.*, 2017 ▸). In the present work, we applied charge-density analysis to the E222Q variant in order to reveal the kind of interactions that effectively regulate the electronic state of the chromophore. The resulting information will enable us to achieve the rational design of new GFP variants and chromophore analogs through quantum-chemical analyses.

## 2. Materials and methods   

### 2.1. Sample preparation   

In this study, the T203I/cycle3 variant (hereafter referred to as T203I) was used in structural analysis for the ‘A’ form, while the S65T/cycle3 (S65T) and E222Q/cycle3 (E222Q) variants were used for the ‘B’ form. The initial gene construct encoding the GFP from *A. victoria* was purchased from the GenScript custom synthesis service (GenScript, Piscataway, New Jersey, USA) and reloaded to a pET-21a plasmid (Novagen, Madison, Wisconsin, USA). This sequence includes the cycle3 mutations (F99S/M153T/V163A; Crameri *et al.*, 1996 ▸) and a His_6_ tag at the C-terminus. The T203I, S65T and E222Q mutations were introduced using the inverse PCR method. The recombinant protein was expressed and purified based on previously reported methods (Palm *et al.*, 1997 ▸; Shinobu *et al.*, 2010 ▸). The transformed *Escherichia coli* BL21(DE3)pLysS cells (Invitrogen, Carlsbad, California, USA) were grown in LB medium with 100 mg ml^−1^ ampicillin and 35 mg ml^−1^ chloramphenicol and were induced at an OD_600_ of ∼0.7 with 1 m*M* IPTG for 16 h at 22°C. The collected cells were mixed with lysate buffer [200 m*M* Tris–HCl pH 8.5 supplemented with BugBuster (Novagen)] and shaken for 24 h at room temperature. The cell extract was purified with an Ni–NTA affinity column (Qiagen, Germantown, Maryland, USA). The His-tag sequence and C-terminal loop were truncated with subtilisin (at a 1:100 ratio to GFP) in 300 m*M* imidazole, 150 m*M* NaCl, 20 m*M* Tris–HCl pH 8.5. The digested protein was purified by anion-exchange chromatography on a Mono Q column (GE Healthcare, Little Chalfont, England) followed by gel filtration on a Superdex 75 column (GE Healthcare). The purified protein was dialyzed against 20 m*M* Tris–HCl pH 8.5. N-terminal amino-acid sequencing (Hokkaido System Science, Sapporo, Japan) and mass spectrometry revealed that the construct contained 230 residues: Ser2–His231. The N-terminal methionine (Met1) may be removed by an enzyme from *E. coli*. The GFP variants were crystallized at 10–15 mg ml^−1^ by the hanging-drop vapor-diffusion method under almost the same conditions as reported previously (Ormö *et al.*, 1996 ▸; Barondeau *et al.*, 2002 ▸). Needle-like crystal clusters were obtained in drops consisting of a mixture of 1 µl precipitant solution (20 m*M* Tris–HCl pH 8.5, 25 m*M* MgCl_2_, 20–30% PEG 4000) and 1 µl protein solution at 20°C. For the S65T and E222Q variants, the initial crystal clusters were crushed, serially diluted in the precipitant solution and used as microseeds to grow single crystals. Large single crystals were then used as macroseeds to grow large crystals at 35°C. Crystals with typical dimensions of 1.5 × 0.3 × 0.3 mm were obtained within 1.5 months. For the T203I variant, large crystals with typical dimensions of 1.0 × 0.1 × 0.1 mm were obtained in almost the same manner as for the other variants but with MES–NaOH pH 5.0 buffer within one month. All crystals were flash-cooled in a nitrogen-gas stream at about 100 K after soaking in a solution consisting of 20 m*M* Tris–HCl pH 8.5, 25 m*M* MgCl_2_, 35–40% PEG 4000 and were stored in a tank of liquid nitrogen until the diffraction experiments.

The purified GFPs were dissolved (at 3–10 µg ml^−1^) in buffer consisting of 100 m*M* NaCl, 10 m*M* MES, 10 m*M* MOPS, 10 m*M* citrate for spectroscopic measurements. Predetermined aliquots of 6 *M* HCl or 4 *M* NaOH were slowly added with rapid stirring to titrate the pH from 4.0 to 8.5 (Kneen *et al.*, 1998 ▸). Absorbance spectra were measured on a V-630 spectrophotometer (JASCO, Hachioji, Japan) at 20°C.

### 2.2. Diffraction data collection for high-resolution analyses   

Diffraction data for T203I and S65T were measured on the BL44XU beamline at SPring-8, Harima, Japan using X-rays of wavelength 0.75 Å. Diffraction data for E222Q were measured on the BL41XU beamline of SPring-8 using high-energy X-rays of wavelength 0.35 Å. The crystals were cryocooled in a helium-gas stream at about 30 or 60 K during data collection. The diffraction intensities for T203I and S65T were recorded using an MX300HE (Rayonix, Evanston, Illinois, USA) detector by the conventional oscillation method and were processed using the *HKL*-2000 program suite (Otwinowski & Minor, 1997 ▸). The diffraction intensities for E222Q were recorded using a PILATUS3 X CdTe 300K detector (Dectris, Baden, Switzerland) which was offset to allow data collection to higher resolution. A total of 7200 images of E222Q diffraction were integrated, scaled and merged with *XDS* (Kabsch, 2010 ▸). The maximum doses for each irradiated position were estimated with *RADDOSE* (Paithankar *et al.*, 2009 ▸) to be 1.7 × 10^4^ Gy for T203I, 9.0 × 10^4^ Gy for S65T and 1.4 × 10^5^ Gy for E222Q. Compared with the reported investigations of the effects of radiation on the spectra of GFP-like proteins (Adam *et al.*, 2009 ▸; Royant & Noirclerc-Savoye, 2011 ▸; Clavel *et al.*, 2016 ▸), the present dose values in our high-resolution analysis were sufficiently low. The experimental conditions and crystallo­graphic statistics are listed in Table 1 ▸.

### 2.3. Evaluation of X-ray damage   

For the assessment of X-ray damage in our study, successive data sets (from 2.0 × 10^4^ to 2.0 × 10^5^ Gy) were measured on the BL41XU beamline at SPring-8. Ten data sets were collected at 50 and 100 K using two positions of the S65T crystal. The X-ray wavelength was set to 0.7 Å, and the diffraction data sets were collected with a PILATUS 6M detector (Dectris). The crystal was cryocooled with a helium-gas stream. The diffraction images were integrated and processed with *XDS*. The resolution limit was 1.2 Å for all 20 data sets (Supplementary Table S1). Structure refinements were carried out with *PHENIX* (Adams *et al.*, 2010 ▸) for all data sets. Difference maps against the first data set with the lowest dose (*F*_o_^*n*th^ − *F*_o_^1st^) were generated using the calculated phase from the structure of the first data set at each temperature. Temperature factors for the three atoms (O_δ1_ of Glu222, Wat2 and Wat4) which show the most significant densities in the difference maps were averaged and the changes were plotted against dose. The data were fitted to a linear function for each temperature.

### 2.4. Structure refinement with the independent spherical atom model   

A crystal structure of the F64L/I167T/K238L GFP variant (PDB entry 2wur; Shinobu *et al.*, 2010 ▸) at 0.90 Å resolution was used as the initial model in the molecular-replacement method. Structure refinement of T203I, S65T and E222Q was initially carried out with *PHENIX*. The geometric restraint for the chromophore group was derived from the geometry of the initial model and was gradually reduced during the course of refinement. All non-H atoms were refined with anisotropic *B* factors. H atoms were added to the model as ‘riding hydrogens’. Some water molecules or magnesium ions were distributed as a cluster. Although they could not be completely modeled with categorization into alternative conformations, they must not be at close distances simultaneously. Some final steps of the refinement were performed with *SHELXL* (Sheldrick & Schneider, 1997 ▸). In this step, the chromophore was not restrained geometrically. The calculation of the estimated standard deviations of bonds was also performed by full-matrix unrestrained refinement in *SHELXL*. H atoms which could not be confirmed in the *F*_o_ − *F*_c_ OMIT map (1.5σ contour level) were removed from the structures. The number of remaining H atoms was 84–91% of all possible H sites. All of the the electron-density maps for the independent spherical atom model (ISAM) were calculated with *PHENIX*. The *R*_work_ and *R*_free_ factors are listed in Table 2 ▸. The determined structures are listed with the previously reported crystal structures of FPs in Supplementary Table S2. Diffraction precision indicator (DPI) values (Cruickshank, 1999 ▸) were calculated for all structures and were used for comparison of the accuracy of these structures.

### 2.5. Charge-density analysis of E222Q with the multipolar atomic model   

Charge-density analysis of E222Q was performed with the multipolar atomic model (MAM) using *MoPro* (Guillot *et al.*, 2001 ▸). The structure factors *F*_o_ were scaled to *F*_c_ in the same manner as described previously (Hirano *et al.*, 2016 ▸). The MAM is expressed as follows (Hansen & Coppens, 1978 ▸):
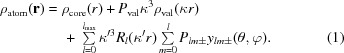
The first two terms describe the spherical core and valence electron densities, and the third term describes the non­spherical distribution of the valence electrons. *P*_val_ and *P*_*lm*±_ are population coefficients. The *R*_*l*_ are Slater-type radial functions and the *y*_*lm*±_ are real spherical harmonic angular functions. Multi-conformational residues, waters without two H atoms and atoms with high temperature factors (*B*_eq_ > 8 Å^2^) were not selected for the MAM refinement. However, the major conformations of Ser65 (*q* = 0.95) and Gln222 (*q* = 0.78) were selected. In total, 39.4% of all atoms were selected for refinement. These included all atoms of the chromophore, His148, Ser205, Gln222 and Wat3, a water hydrogen-bonded to the chromophore. In addition, 13 water molecules with two H atoms were selected. Prior to introducing the initial values of the multipole parameters, higher-order refinement was performed for the selected atoms using data in the resolution range 1.0–0.78 Å without geometric restraints. The initial values of the multipole parameters were then transferred from the ELMAM library (Zarychta *et al.*, 2007 ▸). The initial values for the chromophore were prepared from a theoretical database (Jarzembska & Dominiak, 2012 ▸). The positions of the H atoms were changed to the standard geometry derived from neutron diffraction analyses (Allen, 1986 ▸). The *P*_val_ and *P*_*lm*±_ values were refined in the MAM refinement, while κ and κ′ were fixed to the initial values. The *P*_val_ and *P*_*lm*±_ values were constrained based on the chemical similarities. The *R*_work_ and *R*_free_ factors in the final MAM refinement converged to 11.0% and 13.0%, respectively. Refinement and model statistics are listed in Table 2 ▸. After the MAM refinement, full-matrix refinement was performed to estimate the bond standard deviations. The deformation maps were calculated (Jelsch *et al.*, 2000 ▸) by
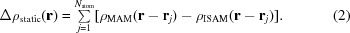



Topological analysis based on the AIM theory was performed with *VMoPro* (Guillot *et al.*, 2001 ▸). The atomic charges based on the atomic boundary defined by the AIM theory were calculated with *BADER* (Yu & Trinkle, 2011 ▸). The bond orders *n*_topo_ were calculated according to a previous report (Tsirelson *et al.*, 2007 ▸). The dissociation energy *D*_e_ of hydrogen bonding was calculated from the density value at the bond critical point (BCP) according to previously published equations (Espinosa *et al.*, 1999 ▸; Espinosa & Molins, 2000 ▸) as follows:
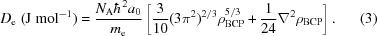
The noncovalent interaction (NCI) surfaces were calculated by a homemade program for reading cube-format electron-density files. The same method as used in *NCIPLOT* (Contreras-García *et al.*, 2011 ▸) was applied for cutoffs. The interaction surface is defined with the isosurface of the reduced density gradient (RDG) as follows:
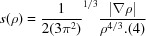
The two-dimensional contour maps were prepared using *VMoPro* and the three-dimensional molecular figures were prepared using *PyMOL* (Schrödinger) or *VMD* (Humphrey *et al.*, 1996 ▸).

## 3. Results   

### 3.1. Crystallographic assessment of X-ray damage   

**Figure 2:**
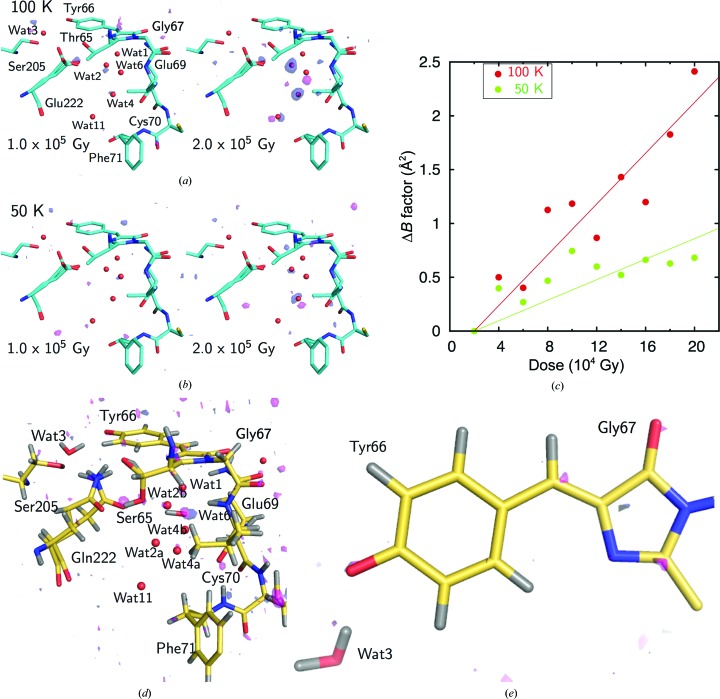
The effect of radiation on the chromophore of GFP. (*a*, *b*) Fourier difference maps of the S65T variant between the initial data set and the irradiated data set at 100 K (*a*) and 50 K (*b*). Positive density contoured at 3.5σ is shown in magenta, while negative density contoured at −3.5σ is shown in dark blue. The occupancy of the minor conformation of Glu222 is 0.25. (*c*) The relationship between the X-ray dose and the change in temperature factor averaged for the specific atoms O_δ1_ of Glu222, Wat2 and Wat4. (*d*, *e*) The Fourier difference map of the E222Q variant between the first and second halves of the data set. The occupancies of the minor conformations of Thr65 and Gln222 are 0.05 and 0.22, respectively. (*d*) is from the same point of view as (*a*) and (*b*), while (*e*) is from a perpendicular direction to the plane of the chromophore. The data sets were collected at 60 K. The cumulative dose of the latter data set was 1.4 × 10^5^ Gy.

By using a series of data sets collected with an increase in the absorption dose, we assessed the influence of X-rays. At 100 K, strong densities were observed at the O_δ1_ atom of Glu222 and two waters (Wat2 and Wat4) in difference maps at more than 1 × 10^5^ Gy [Fig. 2 ▸(*a*)]. Negative peaks were predominant over positive peaks at these atoms. This suggests that only thermal fluctuations are induced by X-ray irradiation and that changes of atomic positions are negligible in this dose range. In addition, difference densities are not observed at the S atoms of methionine and cysteine residues, whereas S atoms are generally sensitive to X-rays (Helliwell, 1988 ▸; Burmeister, 2000 ▸; Garman, 2010 ▸). No specific densities are observed in the region away from the chromophore, as reported previously (Royant & Noirclerc-Savoye, 2011 ▸). The damage that was observed at 100 K was drastically suppressed at 50 K [Fig. 2 ▸(*b*)]. No significant densities were observed in the difference (*F*_o_^*n*th^ − *F*_o_^1st^) maps even at 1.0 × 10^5^ Gy at 50 K. Therefore, we concluded that the intrinsic structural features around the chromophore are maintained up to an X-ray dose of ∼1.0 × 10^5^ Gy at ∼50 K. According to plots of the changes in the temperature factors of the three atoms against the dose, the influence of X-ray irradiation at 50 K was clearly suppressed to less than half of that at 100 K [Fig. 2 ▸(*c*)]. In the data sets used for the crystallographic analysis at ultrahigh resolution, the data set for the E222Q variant, which was collected under the most irradiated condition (1.4 × 10^5^ Gy at 60 K), exhibited no critical structural disturbance in a difference electron-density map calculated with the first (7 × 10^4^ Gy) and second halves (1.4 × 10^5^ Gy) of the data set [Figs. 2 ▸(*d*) and 2 ▸(*e*)]. This result guarantees that radiation damage is negligible even at the X-ray dose. Therefore, we used the full data set in the further structural analysis of E222Q.

### 3.2. X-ray analysis at ultrahigh resolution   

**Figure 3:**
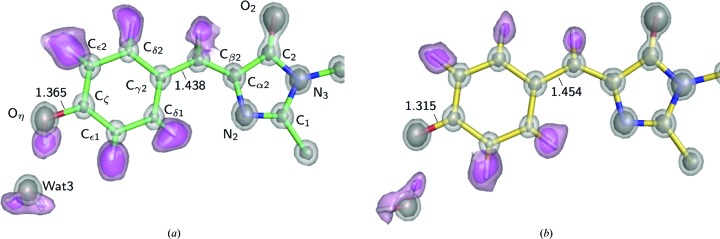
X-ray analysis of GFP from *A. victoria*. (*a*) Electron density for the T203I variant. The 2*F*_o_ − *F*_c_ map contoured at 4σ and 6σ and the *F*_o_ − *F*_c_ OMIT map for H atoms contoured at 2σ and 3σ are shown in gray and pink, respectively. The values shown in the figures are the C_ζ_—O_η_ and C_β2_–C_γ2_ bond lengths in Å. (*b*) Electron density for the E222Q variant.

Diffraction data for all variants were measured at pH 8.5, where the desired forms were contained at ∼90% [Supplementary Fig. S1(*b*)] and where the spectroscopic properties were the same as those at the physiological pH of ∼7.4 for all variants. Crystallographic refinements were performed at resolutions of 0.78–0.94 Å (Tables 1 ▸ and 2 ▸). The electron densities of H atoms revealed that the dual protonation states had been eliminated by means of the amino-acid substitutions (Fig. 3 ▸). Almost all (84–91%) of the H atoms of GFPs were clearly observed in the electron-density maps. The unobserved H atoms were mainly located in multi-conformational residues in the peripheral part of GFP. In addition, the crystallographic analysis revealed twisted peptide bonds with a torsion angle of more than 10° from the planar configuration, which are not usually detected in protein structural analysis [Supplementary Fig. S2(*a*)]. Judging from the deviations of peptide distortion, the amino-acid substitutions did not result in any obvious structural differences except around the chromophore [Supplementary Fig. S2(*b*)]. We thus obtained accurate geometries for GFP in each form. The chromophores showed no apparent tilt or twist along the bridge between the imidazolinone and phenolate rings (Supplementary Table S2). The comprehensive geometric difference between the A and B forms can be seen in the phenolate moiety. The C_ζ_–O_η_ bond in the A form is 1.365 (10) Å, which is 0.05 Å longer compared with those in the B form: 1.314 (7) Å (S65T) or 1.315 (8) Å (E222Q). On the other hand, the imidazolinone ring and the bridging methine group only deviate within the standard error.

### 3.3. Accurate geometric parameters of the chromophore   

**Figure 4:**
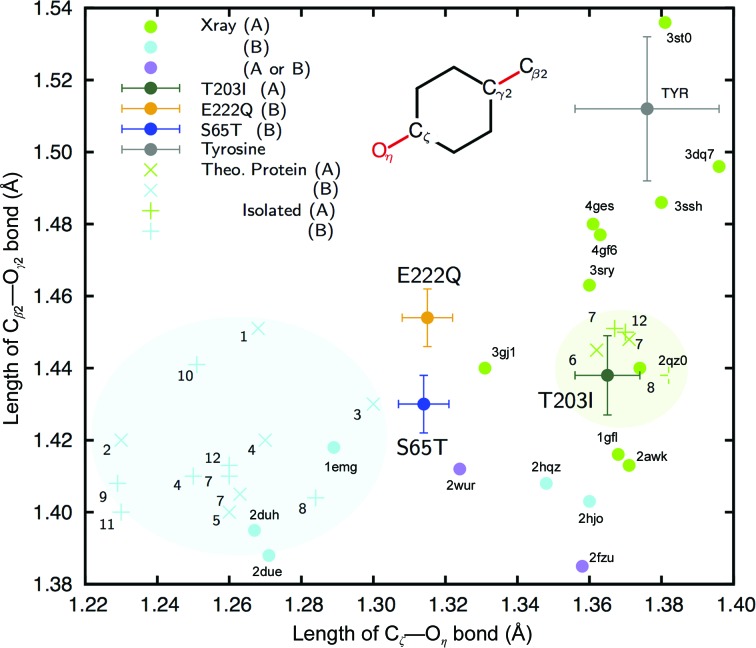
Relationships between the C_ζ_—O_η_ and C_β2_—C_γ2_ bond lengths. Green, cyan and purple circles represent the lengths in GFP variants reported in the Protein Data Bank at higher resolution (<1.3 Å) or referred to in theoretical studies (Supplementary Tables S2 and S3). The proteins are categorized by their reported spectrum or the estimated protonation state of O_η_. Those with an absorption peak at a shorter wavelength (∼400 nm) or a protonated O_η_ are considered to be A-form proteins (green), while those with an absorption peak at a longer wavelength (∼480 nm) or a deprotonated O_η_ are considered to be B-form proteins (cyan). Those with an ambiguous interpretation are shown in purple. The lengths for the structures in this study (T203I in dark green, S65T in blue and E222Q in yellow) are represented with error bars of the standard deviations as estimated with *SHELXL* and *MoPro*. Those of tyrosine are shown in the same manner with the standard deviation for the restraint. Bond lengths optimized by theoretical calculation with or without consideration of the protein environment (‘Theo. Protein’ or ‘Isolated’) are shown as ‘×’ and ‘+’, respectively. The numbers indicate the references listed in Supplementary Table S3.

Computational studies have revealed that the resonance of the bridging bond (C_β2_—C_γ2_) is highly correlated with the charge at O_η_ (Weber *et al.*, 1999 ▸). We therefore made a plot of the C_ζ_—O_η_ and C_β2_—C_γ2_ bond lengths for comparison with various theoretical and crystallographic results (Fig. 4 ▸). Based on their resonances, our currently determined structures and most of the previously reported structures show substantially shorter lengths than in tyrosine for C_β2_—C_γ2_. For the A form, the C_ζ_—O_η_ bond in the structure we determined (T203I) was in good correspondence with the C_ζ_—O_η_ bond in most of the previously reported theoretical and crystal structures. On the other hand, the structures of the B form (S65T and E222Q) differed from almost all of them. The theoretically optimized structures of the A and B forms occupy respective domains in the plot with respect to the length of the C_ζ_—O_η_ bond. The values for the A form indicate that the bonds are single bonds (*d*_C—O_ ≃ 1.38 Å). Those for the B form vary among the previous studies, but overall they indicate a bond longer than a typical double bond (*d*_C=O_ ≃ 1.24 Å). The bonds in our structures indicate that the chromophore of the B form in GFP is intermediate in length between the phenolic and quinone forms. The bond length should be influenced by interactions in the protein environment; however, the theoretical models have not successfully reproduced these interactions even with consideration of the surrounding residues. The GFP structure at 0.90 Å resolution (F64L/I167T/K238N variant; PDB entry 2wur) shows a similar geometry to our structures in the B form, while Tyr66 is protonated in the structure model (Shinobu *et al.*, 2010 ▸). Its interpretation is complicated because the variant has a mixed A/B form in a variable ratio from 0.7 to 1.6 which can be estimated from the dual peaks in its absorption spectrum (Palm *et al.*, 1997 ▸).

### 3.4. Hydrogen-bonding network around the chromophore   

**Figure 5:**
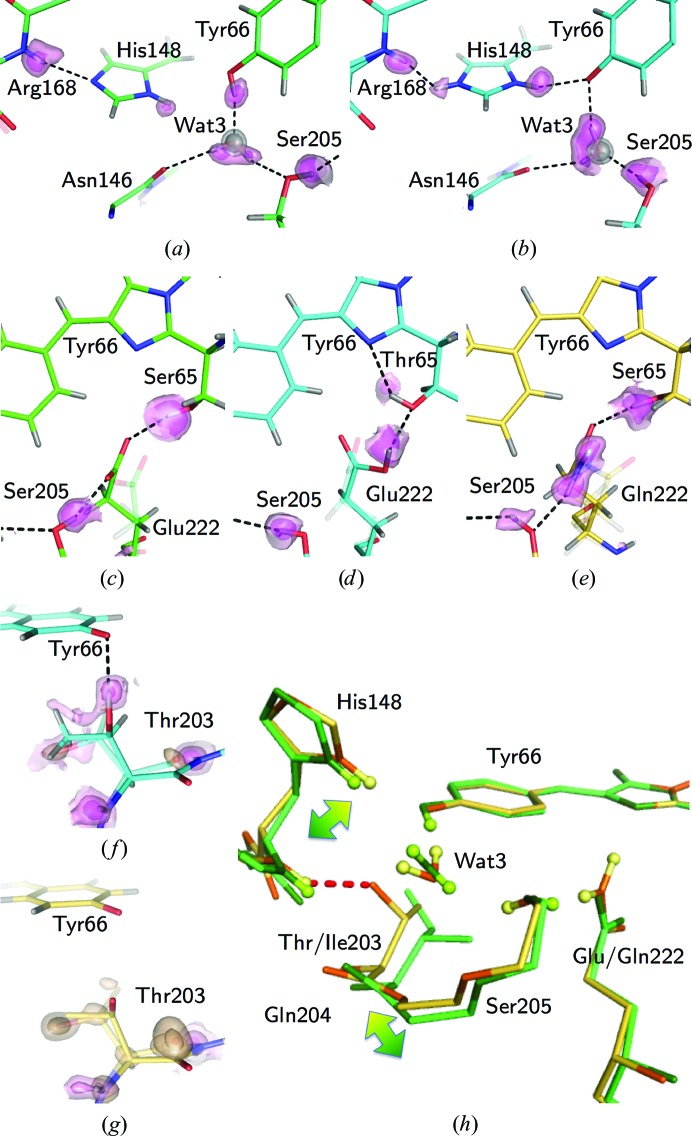
Differences in H-atom positions. (*a*) Electron density of H atoms around His148 and Tyr66 in T203I. (*b*) Electron density of H atoms around His148 and Tyr66 in S65T. (*c*) Electron density of H atoms around Glu222 and Ser65 in T203I. (*d*) Electron density of H atoms around Glu222 and Thr65 in S65T. (*e*) Electron density of H atoms around Gln222 and Ser65 in E222Q. The 2*F*_o_ − *F*_c_ map for Wat3 contoured at 4σ and 6σ and the *F*_o_ − *F*_c_ OMIT map for H atoms contoured at 2σ and 3σ are shown in gray and pink, respectively. Hydrogen bonds are represented as broken lines. The distances between the donor and acceptor are listed in Supplementary Table S4. The minor conformations are shown as transparent models. (*f*) Electron density of H atoms and a minor conformation of Thr203 in S65T. Its occupancy is 0.08. (*g*) Electron density of H atoms and a minor conformation of Thr203 in E222Q. Its occupancy is 0.19. The *F*_o_ − *F*_c_ OMIT map for the minor conformations contoured at 4σ and 7σ is shown in beige. The *F*_o_ − *F*_c_ OMIT map for H atoms contoured at 1σ, 2σ and 3σ is shown in pink. (*h*) The structural difference between the A (T203I) and B (E222Q) forms. The gradient-colored arrows indicate the positional shift between the two forms. A hydrogen bond between Thr203 and His148 in the E222Q variant is shown as a red dotted line, with a donor–acceptor distance of 2.956 (9) Å.

The electronic structure of the chromophore can be greatly affected by the hydrogen bonding to the surrounding residues (Schellenberg *et al.*, 2001 ▸). The H-atom densities in the study provide unambiguous experimental evidence of the hydrogen-bonding patterns in the A and B forms (Fig. 5 ▸). In addition, the visualized protonated states of dissociable residues were consistent with the differences in specific bond lengths and angles [Supplementary Figs. S3(*a*)–S3(*h*)]. For the H atoms, the most prominent differences between the A and B forms around the chromophore are the protonation states of His148 and Glu222. His148 is neutral (deprotonated) in the A form [Fig. 5 ▸(*a*)], while it is positively charged (protonated) in the B form [Fig. 5 ▸(*b*)]. This charge may electrostatically stabilize the negative charge of the chromophore at O_η_.

Two H atoms of Wat3, which is bound to O_η_ of the chromophore, were observed in both forms. One H atom of Wat3 interacts with Ser205 in the A form, while it interacts with O_η_ in the B form [Supplementary Figs. S3(*i*) and S3(*j*)]. The other H atom interacts with the main-chain carbonyl of Asn146. This hydrogen bond had been obscure in previous research because of the absence of electron density for the H atoms of Wat3 (Brejc *et al.*, 1997 ▸; Cubitt *et al.*, 1995 ▸; Shinobu *et al.*, 2010 ▸). Our electron densities clearly showed this interaction in both forms. The difference in the orientation of Wat3 leads to a knock-on effect on the hydrogen-bonding network between O_η_ of the chromophore and O_γ_ of Ser205. On the other hand, the hydrogen-bonding patterns around Ser/Thr65 and Glu/Gln222 are significantly different [Figs. 5 ▸(*c*), 5 ▸(*d*) and 5 ▸(*e*)], while both S65T and E222Q are in the B form. The hydroxyl H atom of Thr65 in S65T is hydrogen-bonded to N_2_ of the imidazolinone ring of the chromophore, while the corresponding H atom of Ser65 in E222Q is hydrogen-bonded to Gln222. This implies that the hydrogen bonds around Ser/Thr65 and Glu/Gln222 are not directly critical for the protonation state of the chromophore. On the other hand, the negative charge at Glu222 is correlated with the protonation state of O_η_ of the chromophore through the orientation of the hydroxyl H atom of Ser205. While this mechanism has generally been accepted (Brejc *et al.*, 1997 ▸; Palm *et al.*, 1997 ▸), we experimentally confirm it with the electron density of H atoms.

The electron densities for the hydroxyl H atom of Thr203 reveal that the atomic position shows a double conformation or disordered state in the S65T and E222Q variants, in which the B form is dominant [Figs. 5 ▸(*f*) and 5 ▸(*g*)]. The side chain of Thr203 is hydrogen-bonded to the main chain of His148 only in the B form [Fig. 5 ▸(*h*)]. This interaction causes positional differences in residues Thr203–Ser205, and it may thereby influence the protonation states of His148, Glu222 and the chromophore.

### 3.5. Charge-density information   

**Figure 6:**
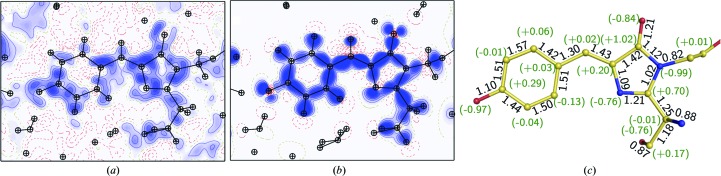
Charge-density analysis of the E222Q variant. (*a*) The residual density in a slice of the chromophore plane after ISAM refinement. (*b*) The static deformation density in a slice of the chromophore plane after MAM refinement. The contour intervals are 0.05 e Å^−3^. Positive, blue lines; negative, red broken lines. The intervals are only colored in blue. (*c*) AIM charge values are shown in parentheses on each atom, while topological bond orders are given along each covalent bond. H atoms and their positive charges are not shown.

**Figure 7:**
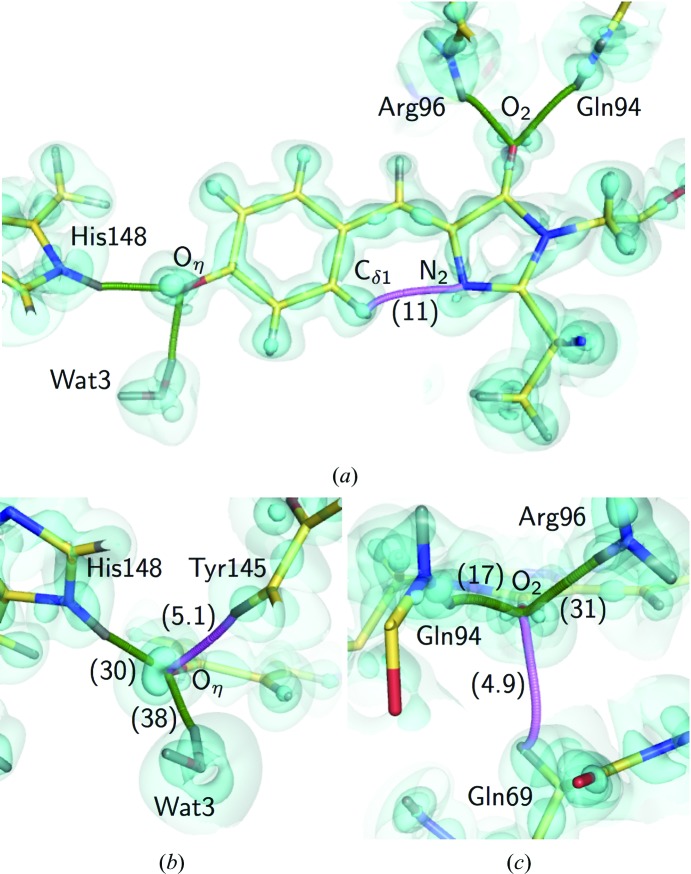
The hydrogen bonding based on the MAM. (*a*) The bond paths of hydrogen bonding along the electron distribution of the chromophore. (*b*) Details of the bond paths around O_η_. (*c*) Details of the bond paths around O_2_. The cyan surfaces represent the deformation electron density at contour levels of +0.01, +0.15 and +0.5 e Å^−3^, respectively. The bond paths for conventional hydrogen bonds are represented as green curves, while those for nonconventional bonds are represented as pink curves. The *D*_e_ values for hydrogen bonds (in kJ mol^−1^; listed in Supplementary Table S5) are shown in parentheses.

For the E222Q variant in the B form, we performed MAM refinement because residual electron densities were clearly observed between covalently bonded atoms and in the non­bonded direction around O or N atoms in the *F*_o_ − *F*_c_ map, even after the conventional ISAM refinement [Fig. 6 ▸(*a*), Supplementary Fig. S4]. They disappeared after the MAM refinement, proving the success of the refinement. The valence electrons are sufficiently identified in the MAM electron density [Fig. 6 ▸(*b*)]. We applied a topological analysis based on the atoms-in-molecules (AIM) theory (Bader, 1990 ▸) for quantification of the individual interactions involving the chromophore [Supplementary Figs. S5(*a*) and S5(*b*)]. The atomic charge and bond orders are shown in Fig. 6 ▸(*c*). The phenolic oxygen (O_η_) has a negative charge (−0.97), which has been thought to be stabilized by hydrogen bonds. The carbonyl O atom in the imidazolinone moiety (O_2_) is also comparably negative (−0.84). Consequently, the total negative charge of the chromophore (−1.05) is allocated to both the phenolate and imidazolinone moieties. The MAM electron density was also used to estimate a topological bond order for the individual covalent bonds. The bond lengths and orders show an approximately linear relationship for each type of bond, C—C, C—O and C—N [Supplementary Fig. S5(*c*)], as expected. The C_ζ_—O_η_ bond is clearly distinct from all of the other C—O bonds, while the C_2_–O_2_ bonds are only slightly different from other carbonyl bonds of the main chain. The estimated bond order of C_2_—O_2_ is slightly lower than the order of the other carbonyl bonds of the main chain, despite the comparable bond length. This weakened C_2_—O_2_ bond is consistent with the red shift previously reported in an IR spectrum (Stoner-Ma *et al.*, 2006 ▸). We detected the hydrogen bonds and estimated the dissociation energy (*D*_e_) from the topological parameters for the hydrogen bonds between the chromophore and the protein environment. The *D*_e_ values were highly correlated with the length of the hydrogen bonds, as in small molecules [Supplementary Fig. S5(*d*)]. O_η_ was shown to form conventional hydrogen bonds to Wat3 and His148, drawing the bond paths along the electron distribution [Fig. 7 ▸(*a*)]. The stronger interaction between O_η_ and Wat3 (38 kJ mol^−1^) was realized with a linearly aligned lone pair and the water H atom [Fig. 7 ▸(*b*)]. The interactions of O_2_ in the imidazolinone moiety with Gln94 and Arg96 were a little weaker than those of O_η_ [17 and 31 kJ mol^−1^; Fig. 7 ▸(*c*)], in accordance with the long hydrogen-bonding distances (Supplementary Table S5). The interactions of O_2_ were under some tension because the peptide planes of Gln94 and Arg96 were extraordinarily distorted [Supplementary Figs. S2(*c*) and S2(*d*)]. We also observed additional bond paths for the non­conventional hydrogen bonds between O_η_ and Tyr145, O_2_ and Gln69, and phenolate and imidazolinone. These bonds are quite weak (5.1 and 4.9 kJ mol^−1^) and can easily loosen (Daday *et al.*, 2015 ▸), while such interactions may be potential pathways of photoinduced electron transfer in GFP (Bogdanov *et al.*, 2016 ▸). The nonconventional hydrogen-bond bridging between two ring moieties had a *D*_e_ value of 11 kJ mol^−1^ and may contribute to the planarity of the two ring moieties.

### 3.6. Nonbonded interactions around the chromophore   

**Figure 8:**
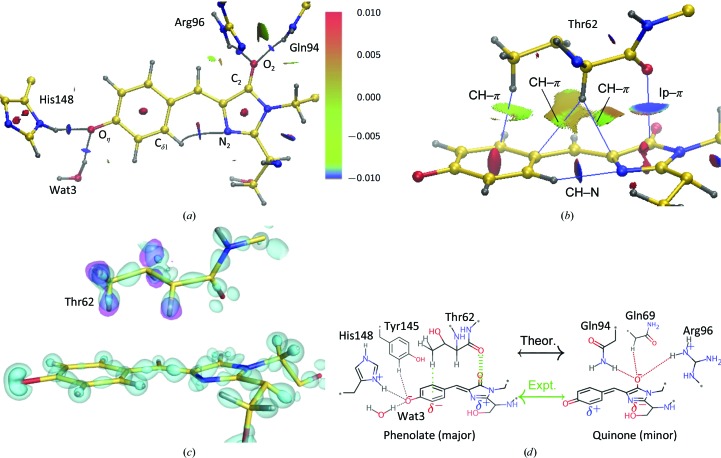
Noncovalent interactions around the chromophore. (*a*) Distribution of the noncovalent interaction surface around the chromophore derived from the MAM. The bond paths for hydrogen bonds are represented as gray curves. The reduced density gradient [*s*(ρ) = 0.4] isosurface is colored according to a blue–green–red scale over the range −0.01 < sign(λ_2_)ρ < +0.01 e *a*_0_^−3^. λ_2_ and *a*_0_ are the second eigenvalue of the Hessian and the Bohr radius, respectively. Blue indicates attraction, green indicates very weak attraction and red indicates repulsion. (*b*) A side view of the interaction surfaces. The abbreviation ‘lp’ is short for ‘lone pair’. (*c*) The distribution of interacting electrons in the corresponding region to (*b*). The cyan surface represents the deformation electron density at contour levels of +0.2 and +0.5 e Å^−3^. The pink surface represents the *F*_o_ − *F*_c_ OMIT map for H atoms of Thr62 contoured at 3.5σ. (*d*) A schematic representation of the resonance structure of the anionic chromophore. Interactions stabilizing each resonance state are indicated. The preceding theoretical calculations predicted that the quinone form is favored (Fig. 4 ▸) or that both states are almost equivalent (Martin *et al.*, 2004 ▸; Petrone *et al.*, 2013 ▸), while the phenolate form was predominant in our experiments.

Next, based on the determined electron density of the E222Q variant, we surveyed the weaker interactions on the chromophore by the noncovalent interaction (NCI) analysis method (Johnson *et al.*, 2010 ▸). The conventional interactions described above can also be identified in this NCI analysis [Fig. 8 ▸(*a*)]. An additional interaction between the C_2_ atom and the carbonyl O atom of Thr62 can be regarded as a lone pair–π* interaction [Figs. 8 ▸(*b*) and 8 ▸(*c*)] (Choudhary *et al.*, 2012 ▸), which actually weakens the C_2_–O_2_ bond, as indicated from the bond order shown in Supplementary Fig. S5(*c*). From the perspective of the imidazolinone, this interaction can also be interpreted as a charge transfer from the lone pair to the π orbital of the ring system.

## 4. Discussion   

In this study, we determined X-ray structures of GFP with the two different protonated states of the chromophore with high accuracy. These structures were obtained using sufficiently low X-ray doses to avoid significant radiation damage. The chromophores are almost completely planar, as expected for FPs in their ground state (Weber *et al.*, 1999 ▸). Therefore, the geometries in this study are plausible, like those in the ground state. The C_ζ_—O_η_ bond lengths in our structure in the B form are distinct from those in previously reported crystal structures. In addition, the theoretically optimized chromophores of the B form are also different from our structures. The previously reported crystal structures were determined under geometric restriction at ordinary resolution and are more or less influenced by the geometric restraints. Conversely, the quantum-chemical calculations are strongly biased by the initial geometry, while the crystal structures are used after energy optimization. For example, PDB entry 1ema, which is one of the first crystal structures of GFP (Ormö *et al.*, 1996 ▸), has a C_ζ_—O_η_ bond length of 1.48 Å. This value is much longer than the lengths in the other structures shown in Fig. 4 ▸, while the crystal structure has been used as an initial model in some theoretical calculations of the chromophore. From a practical standpoint, these two problems are interrelated. The C_ζ_—O_η_ bond length in particular is related to the electronic state of the chromophore (Amat & Nifosì, 2013 ▸; Daday *et al.*, 2015 ▸). Since the chromophore structures in this study were finally determined without restraints, they should be considered to be authentic and suitable for discussing the inter­actions regulating the electronic state of the chromophore.

Although both the S65T and E222Q variants have spectroscopic features of the chromophore in the B form, the hydrogen bonds between Thr203 and the chromophore appear to differ. This indicates that this hydrogen bond is not indispensable in the B form, while this interaction has been considered to be critical for stabilizing the negative charge at O_η_ (Cubitt *et al.*, 1995 ▸). A computational study also stated that this hydrogen bond does not lead to energy lowering in the B form (Grigorenko *et al.*, 2013 ▸), while the optimized geometric parameters around the chromophore were not consistent with our results (Fig. 4 ▸, Supplementary Table S3). Some minor differences could arise from the existence or non-existence of the hydrogen bond between Thr203 and O_η_. The wavelength, intensity and shoulder of the absorption peak in fact differ from each other only slightly, while both have features of the B form. Instead, the conserved hydrogen bond between Thr203 and His148 may influence the protonation states of His148, Glu222 and the chromophore. This hydrogen bond should also be realized in wild-type GFP by rotation of the side chain of Thr203 (Brejc *et al.*, 1997 ▸). This implies that the hydrogen bonding between Thr203 and His148 is the key interaction in the difference between the A and B forms, whereas the interaction between Thr203 and O_η_ of the chromophore has been widely accepted (Remington, 2011 ▸).

The protonation of His148 in the B form is reported for the first time in this study; it was previously predicted for the Y66H/Y145F variant (BFP; Wachter *et al.*, 1997 ▸). It has been demonstrated that the side chain of His148 swings away from the chromophore, thus avoiding the electrostatic repulsion between NH of the just adjacent Arg168 and His148 at acidic pH (Shinobu *et al.*, 2010 ▸). Accordingly, the proton between His148 and Arg168 would not coexist but would be shared between the two residues at physiological pH, as reported for cholesterol oxidase (Lyubimov *et al.*, 2006 ▸). The interaction between His148 and the chromophore may not be very strong, because the positive charge of His148 should stabilize both the anionic chromophore and the partially deprotonated amide of Arg168. Although His148 could feasibly shuttle a proton from the bulk solution (Shinobu *et al.*, 2010 ▸; Agmon, 2005 ▸), this function cannot be realized in the B form because of the break in the hydrogen-bond network from Glu/Gln222. The hydrogen bond between His148 and Arg168 could be interpreted as standing by to participate in the proton transfer, because the immediate proton transfer from the bulk solution is responsible for relaxation. The reduced fluorescence efficiency on dimerization (Agmon, 2005 ▸) or in a redox-sensitive variant (Hanson *et al.*, 2004 ▸) can be considered to result from blocking of the surface region near His148. These findings can be validated by neutron crystallography, which provides native structures that are free from radiation damage (Ashkar *et al.*, 2018 ▸).

The negative charges determined at both O_η_ and O_2_ of the chromophore of the E222Q variant indicate that the chromophore is in a resonant structure between the phenolate and quinone forms. Such charge distributions are mostly consistent with the theoretical model of isolated chromophores (Martin *et al.*, 2004 ▸; Petrone *et al.*, 2013 ▸), but the charge of the phenolic moiety in our observations was slightly more negative than that of the imidazolinone moiety. The lone pair–π interaction between Thr62 and the imidazolinone moiety is simultaneously recognized from the charge-density analysis. Although a lone pair–π interaction is essentially weak, the depletion of electrons in the π orbital enhances the inter­action, which is usually realized by the substitution of electrophilic groups such as halogens on the aromatic ring (Lu *et al.*, 2012 ▸). Conversely, the lone pair–π interaction induces electron delocalization from the aromatic ring to the substituent. In the GFP chromophore, the phenolate moiety acts as an electrophilic group that removes electrons from the imidazolinone. As a result, the resonance of the chromophore is more biased to the phenol form than previously predicted.

This charge dispersion between the two rings enhances the transition moment in the excitation reaction for fluorescence (Drobizhev *et al.*, 2015 ▸). The hydrogen bond between Thr203 and O_η_ has conventionally been considered to be responsible for this charge dispersion; however, this hydrogen bond would not be substantial, as described above. The positive charge of His148 would also not strongly affect the chromophore, because it mainly couples with the partly deprotonated main-chain amide of Arg168. Based on our present findings, we propose that the lone pair–π interaction between Thr62 and the imidazolinone moiety is critical for the charge distribution in the chromophore [Fig. 8 ▸(*d*)]. We could interpret this finding as resulting from an electron donation to the phenolic π orbital from the imidazolinone moiety. For the phenolic group, a weaker CH–π-type interaction with the methyl group of Thr62 was detected, indicating the presence of the attractive dispersion force. On the other hand, the ISAM electron density overestimates the strength of this CH–π-type interaction (Supplementary Fig. S6). This is consistent with the amino acid at this position in FPs being evolutionally variable even among the Hydrozoa and Anthozoa (Supplementary Fig. S7) and the methyl group is not conserved. Most amino acids that will not clash with other residues or the chromophore seem to be able to substitute. Since the variants that we used here are not natural, the authentic interactions in the wild type should be estimated. However, our interpretation does not conflict with the conservation, at least. Thus, we conclude that charge-density analyses enable us to recognize weak but significant intramolecular inter­actions.

The geometries of the chromophores were substantially different from those in the previously reported crystal structures. This may be owing to inadequate geometric restraints having been applied in the crystallographic refinement calculations in the previous analyses. Another difference was found in comparisons with quantum-chemically optimized structures of the B form. This may be owing to the influence of the initial geometry. On the other hand, this also indicates that previous computational models might not completely describe the electronic features of the chromophore in the protein environment. Hydrogen bonds have been mentioned in these models owing to their ability to elucidate the various reactions in proteins. However, our charge-density information indicated that various types of closed-shell interactions (Neel *et al.*, 2017 ▸; Nishio *et al.*, 2014 ▸) exist between the chromophore and the protein environment. Unfortunately, despite their importance, these interactions were not sufficiently considered in the previously reported quantum-chemical calculations. Thr62 was only included in the case of theoretical geometry optimizations for the chromophore (Amat & Nifosì, 2013 ▸; Supplementary Table S3). In the calculation, however, the differences from our anionic chromophore were large, like those in other theoretical studies (Fig. 4 ▸). Since the inter­actions between Thr62 and the chromophore are based on the dispersion force, current approximation methods may not be suitable for the situation in GFP (Neel *et al.*, 2017 ▸). The subatomic resolution structural features of the GFP chromophore in the protein environment were elucidated for the first time by our experimental analyses, since these features are currently unobtainable from computational studies alone. Therefore, ultrahigh-resolution X-ray crystallography should be developed for use in a complementary fashion with quantum chemistry in order to reveal experimentally the elusive electronic structures of proteins.

## 5. Related literature   

The following references are cited in the supporting information for this article: Altoè *et al.* (2005 ▸, 2007 ▸), Beerepoot *et al.* (2013 ▸), Bravaya *et al.* (2011 ▸), Ding *et al.* (2013 ▸), Filippi *et al.* (2009 ▸), Fisher *et al.* (2012 ▸), Olsen & Smith (2008 ▸), Sinicropi *et al.* (2005 ▸), Tozzini & Nifosì (2001 ▸) and Wiehler *et al.* (2003 ▸).

## Supplementary Material

PDB reference: GFP, F99S/M153T/V163A/T203I variant, 6jgh

PDB reference: S65T/F99S/M153T/V163A variant, 6jgi

PDB reference: F99S/M153T/V163A/E222Q variant, 6jgj

Supplementary Figures and Tables. DOI: 10.1107/S205225251900246X/mf5031sup1.pdf

Restraint cif file for T203I and E222Q mutants. DOI: 10.1107/S205225251900246X/mf5031sup2.txt

Restraint cif file for S65T mutant. DOI: 10.1107/S205225251900246X/mf5031sup3.txt

Diffraction project datasets 6jgh: https://doi.org/10.18430/m36jgh

Diffraction project datasets 6jgi: https://doi.org/10.18430/m36jgi

Diffraction project datasets 6jgj: https://doi.org/10.18430/m36jgj

## Figures and Tables

**Table 1:** Data-collection and crystallographic statistics Values in parentheses are for the highest resolution shell.The raw diffraction images are available at the Integrated Resource for Reproducibility in Macromolecular Crystallography (http://proteindiffraction.org/).

	T203I	S65T	E222Q
Mutation	F99S/M153T/V163A/T203I	S65T/F99S/M153T/V163A	F99S/M153T/V163A/E222Q
Data collection			
Crystal size (mm)	0.9 × 0.1 × 0.1	1.3 × 0.3 × 0.3	1.0 × 0.5 × 0.5
Beamline	BL44XU	BL44XU	BL41XU
Detector	MX300HE	MX300HE	PILATUS3 X CdTe 300K
Wavelength (Å)	0.75	0.75	0.35
Temperature (K)	30	60	60
Oscillation angle (°)	0.5	0.5	0.05
Total No. of frames	360	360	7200
Dose per frame (Gy)	8.5 × 10^2^	4.5 × 10^3^	1.9 × 10
Total dose per position (Gy)	1.7 × 10^4^	9.0 × 10^4^	1.4 × 10^5^
Crystallographic data			
Space group	*P*2_1_2_1_2_1_	*P*2_1_2_1_2_1_	*P*2_1_2_1_2_1_
*a*, *b*, *c* (Å)	50.64, 62.51, 68.18	50.86, 62.41, 69.17	50.89, 62.25, 68.86
Resolution (Å)	50.0–0.94 (0.96–0.94)	50.0–0.85 (0.86–0.85)	50.0–0.78 (0.79–0.78)
Total No. of reflections	1263282	1095997	3023703
No. of unique reflections	141942	188965	254665
*R*_merge_ † (%)	14.7 (126.0)	8.6 (111.8)	7.1 (188.2)
〈*I*/σ(*I*)〉	14.8 (1.4)	16.6 (1.5)	16.2 (1.3)
Completeness (%)	99.8 (99.3)	97.8 (98.0)	99.2 (97.6)
Multiplicity	8.9 (7.1)	5.8 (5.3)	12.0 (10.0)
CC_1/2_ (%)	(54.2)	(49.8)	(58.9)
Wilson *B* factor (Å^2^)	5.88	6.36	6.81

† *R*_merge_ = 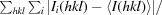 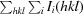.

**Table 2:** Refinement statistics

	T203I	S65T	E222Q
Mutation	F99S/M153T/V163A/T203I	S65T/F99S/M153T/V163A	F99S/M153T/V163A/E222Q
Resolution (Å)	29.93–0.94	46.33–0.85	31.14–0.78
No. of reflections	141832	188950	254639
*R*_work_ †/*R*_free_ ‡ (%)			
ISAM	10.7/12.9	9.2/11.2	11.4/13.0
MAM	—	—	10.8/12.5
No. non-H atoms§			
Protein	1817.7	1810.1	1789.7
Ion	0.7 [Cl^−^]	0	0.8 [Mg^2+^]
Water	461.1	521.0	467.1
No. of H atoms			
Protein	1516.2	1565.8	1357.8
Water	34.3	64.9	23.1
Average temperature factor (Å^2^)			
Protein	7.9	8.2	8.8
Ion	11.8 [Cl^−^]	—	12.9 [Mg^2+^]
Water	20.8	21.1	21.6
Mean anisotropy¶			
Protein	0.35	0.50	0.46
Ion	0.47 [Cl^−^]	—	0.29 [Mg^2+^]
Water	0.34	0.38	0.31

† *R*_work_ = 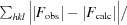 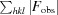.

‡ *R*_free_ was calculated by using 5% of the reflections that were not included in the refinement as a test set.

§ Calculated as the sum of occupancies.

¶ Anisotropy is defined as the ratio of the smallest to the largest eigenvalue of the anisotropic displacement parameter matrix.
