# Supplementary material for: Subatomic resolution X-ray structures of green fluorescent protein
Source: IUCrJ. 2019 Apr 3;6(Pt 3):387–400. doi: 10.1107/S205225251900246X (PMC6503917; doi:10.1107/S205225251900246X)
Supplement: Supplementary file 1 [file m-06-00387-sup1.pdf]

IUCrJ

Volume 6 (2019)

Supporting information for article:

Sub-atomic X-ray structures of green fluorescent protein

Kiyofumi Takaba, Yang Tai, Haruhiko Eki, Hoang-Anh Dao, Yuya Hanazono, Kazuya Hasegawa, Kunio Miki and Kazuki Takeda

Supporting Information for

Sub-atomic X-ray structures of green fluorescent protein

Kiyofumi Takaba[1], Yang Tai[1], Haruhiko Eki[1], Hoang-Anh Dao[1], Yuya Hanazono[1], Kazuya Hasegawa[2], Kunio Miki[1], Kazuki Takeda[1]*

[1]Department of Chemistry, Graduate School of Science, Kyoto University, Sakyo-ku, Kyoto 606-8502, Japan

[2]Protein Crystal Analysis Division, Japan Synchrotron Radiation Research Institute (JASRI), 1-1-1 Kouto, Sayo-cho, Sayo-gun, Hyogo 679-5198, Japan

*Corresponding author. Email: ktakeda@kuchem.kyoto-u.ac.jp

This file includes:

Figure S1 to S7
Table S1 to S5

Two additional electronic files used for the initial refinement stage are also attached as supplement.

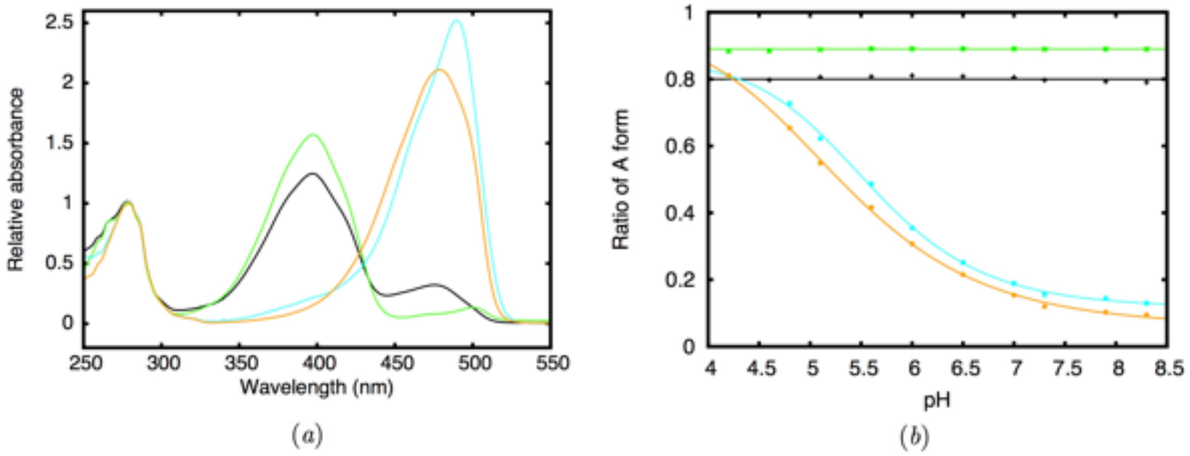

Figure S1

Absorption spectra of GFP variants. (*a*) Absorption spectra of GFP variants at pH 8.5 (black: WT; green: T203I; cyan: S65T; yellow: E222Q). (*b*) Population ratios of the A form at different pH. They were calculated as reported previously (Wiehler *et al.*, 2003). The pKa values from the change of the population in the two anionic variants are 5.58 (S65T) and 5.27 (E222Q), respectively.

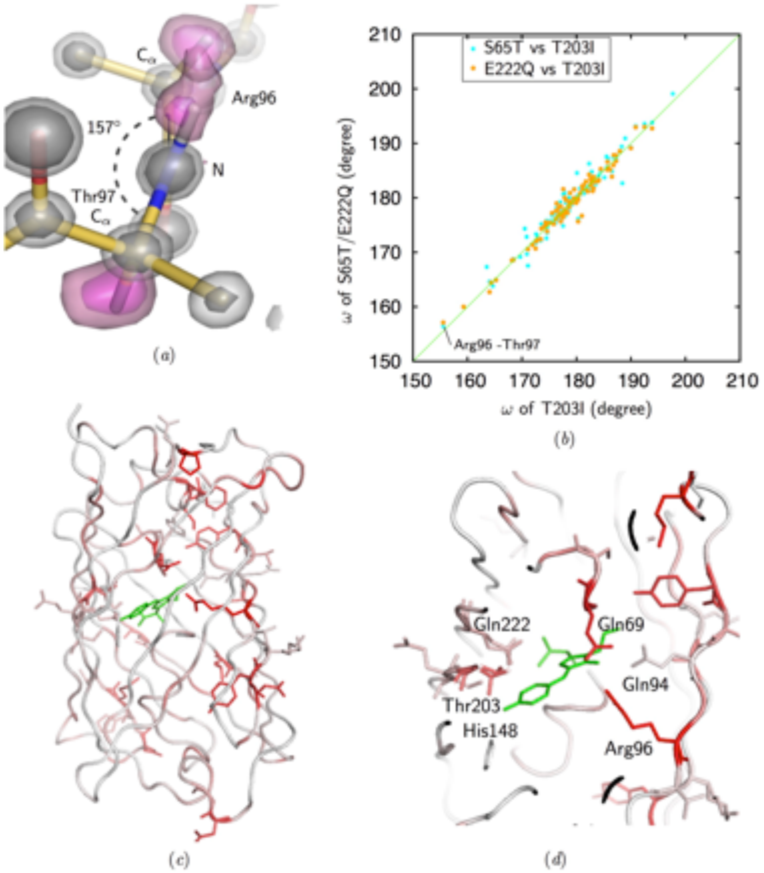

Figure S2

Peptide-bond distortions of GFP. (*a*) Distortion of the peptide bond of Arg96-Thr97 with an ω angle of 157°. The $2F_o$–F_c map contoured at 4σ and 6σ and the F_o–F_c omit map for H-atoms contoured at 2σ and 3σ are shown in grays and pinks, respectively. (*b*) Relationships of the distortion angle of each residue between the variants. The values for the T203I variant are along the fast axis and those of S65T (cyan) and E222Q (yellow) are plotted. The residues whose occupancy of the main conformation is less than 0.7 are ignored. (*c*) Overall structure of the GFP E222Q variant colored by the distortion of peptide bonds. The models are colored according to the absolute values of torsion angles (ω: C_α–C–N'–C_α') from 160° (red) to 180° (white). The highly-distorted residues and the residues interacting with the chromophore are shown in a stick model with side-chains. The chromophore is highlighted in green. (*d*) Close-up view around the chromophore.

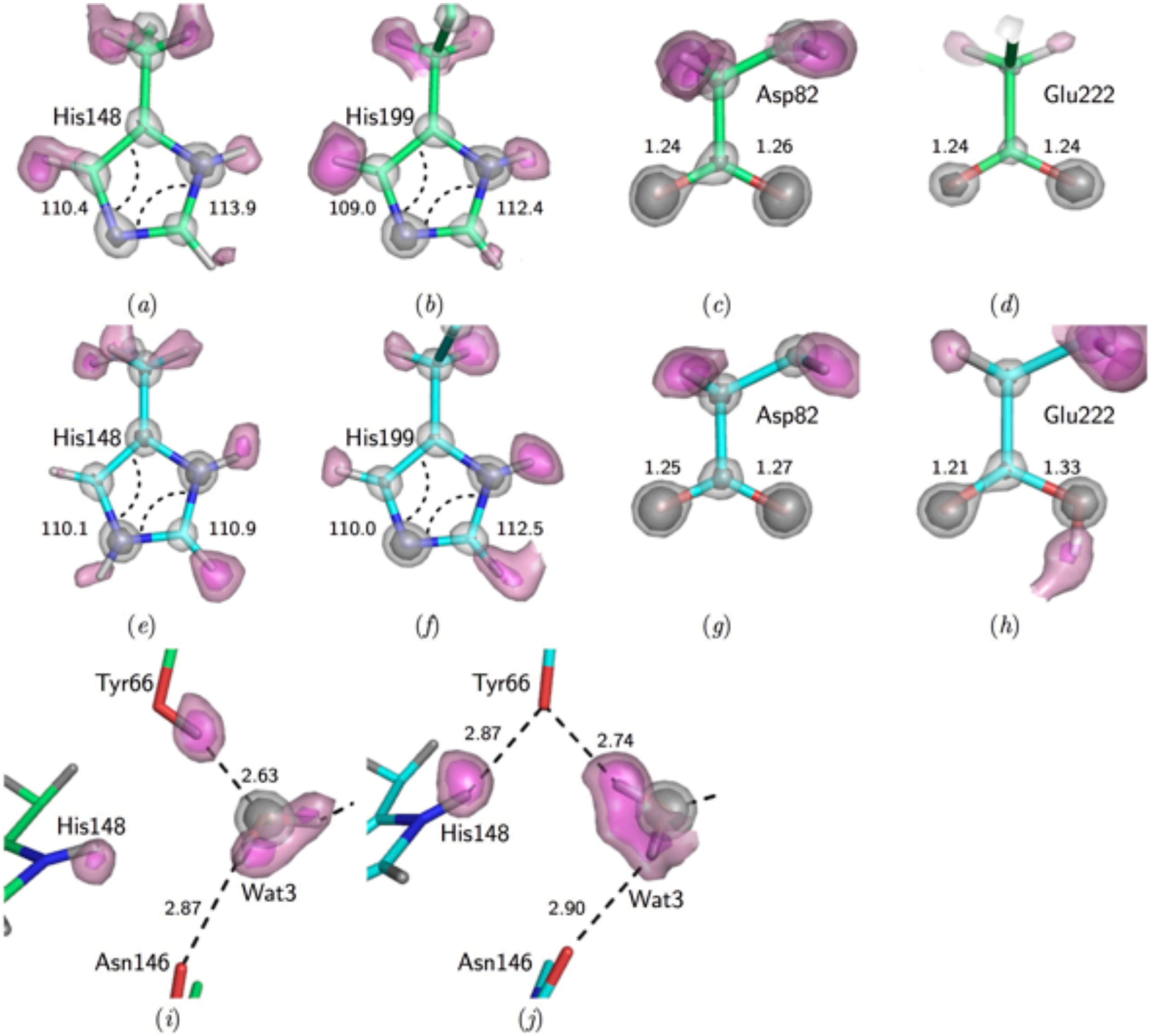

Figure S3

H-atom positions and geometry of dissociable residues and H-bonds around O_η. (*a-h*) Electron density of H-atoms at His148 for the T203I variant (*a*), at His199 for the T203I variant (*b*), at Asp82 for the T203I variant (*c*), at Glu222 for the T203I variant (*d*), at His148 for the S65T variant (*e*), at His199 for the S65T variant (*f*), at Asp82 for the S65T variant (*g*) and at Glu222 for the S65T variant (*h*). The values are bond angles of C_γ–$C_{\delta2}$–$N_{\varepsilon2}$ and $N_{\varepsilon2}$–$C_{\varepsilon1}$–$N_{\delta1}$ of His and lengths of C_γ/C_ε–$O_{\delta1}$/$O_{\varepsilon1}$ and C_γ/C_ε–$O_{\delta2}$/$O_{\varepsilon2}$ of Asp/Glu. These visualized protonation state are consistent with the difference in the bond angles and lengths (Fisher *et al.*, 2012). (*i*, *j*) Close-up view of H-bonds around His148-Wat3 for T203I (*i*) and around

His148-Wat3 for S65T (*j*). The $2F_o-F_c$ map for Wat3 contoured at 4σ and 6σ and the F_o-F_c omit map for H-atoms contoured at 2σ and 3σ are shown in grays and pinks, respectively. H-bonds are represented as broken lines, with the distances between the donor and the acceptor shown in angstroms.

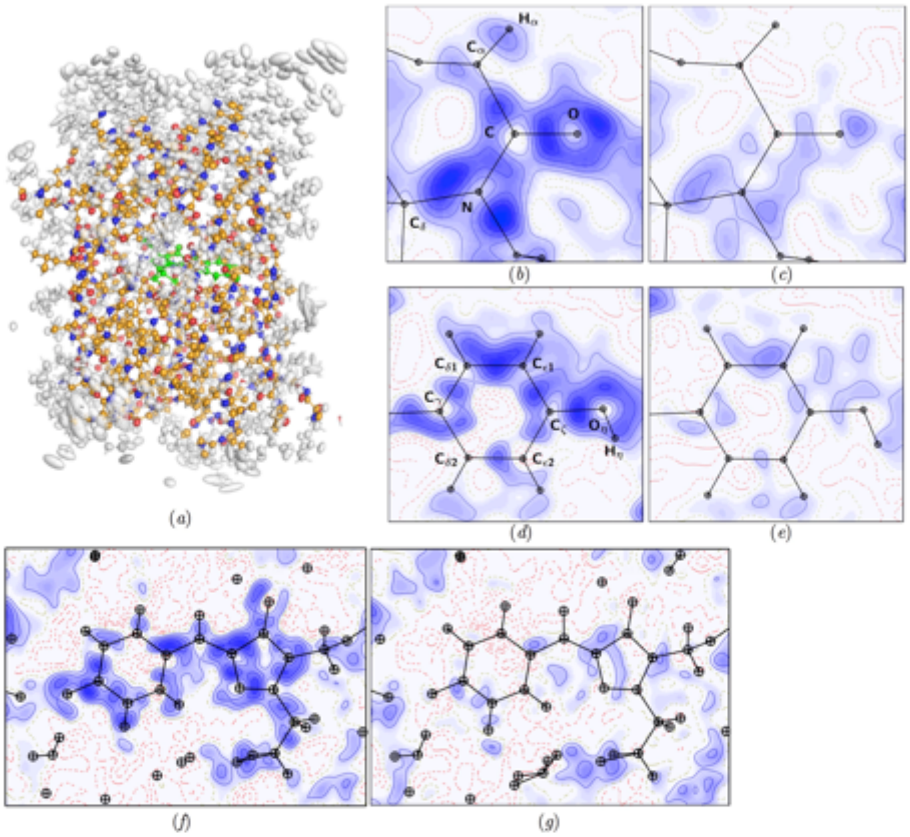

Figure S4

MAM of the E222Q GFP. (*a*) A ball and stick model of the whole GFP. Atoms refined with MAM are colored, while the other fixed atoms are shown in gray. (*b*) The residual density in a slice of the peptide plane between Trp57-Pro58 after ISAM refinement. (*c*) The residual density in a slice of the peptide plane between Trp57 and Pro58 after MAM refinement. (*d*) The residual density in a slice of the phenolic plane of Tyr106 after ISAM refinement. (*e*) The residual density in a slice of the phenolic plane of Tyr106 after MAM refinement. (*f*) The residual density in a slice of the chromophore plane after ISAM refinement. (*g*) The residual density in a slice of the chromophore plane after MAM refinement. The contour intervals are 0.05 e/Å^3. Positive: blue lines; negative: red broken lines. The intervals are only colored in blue.

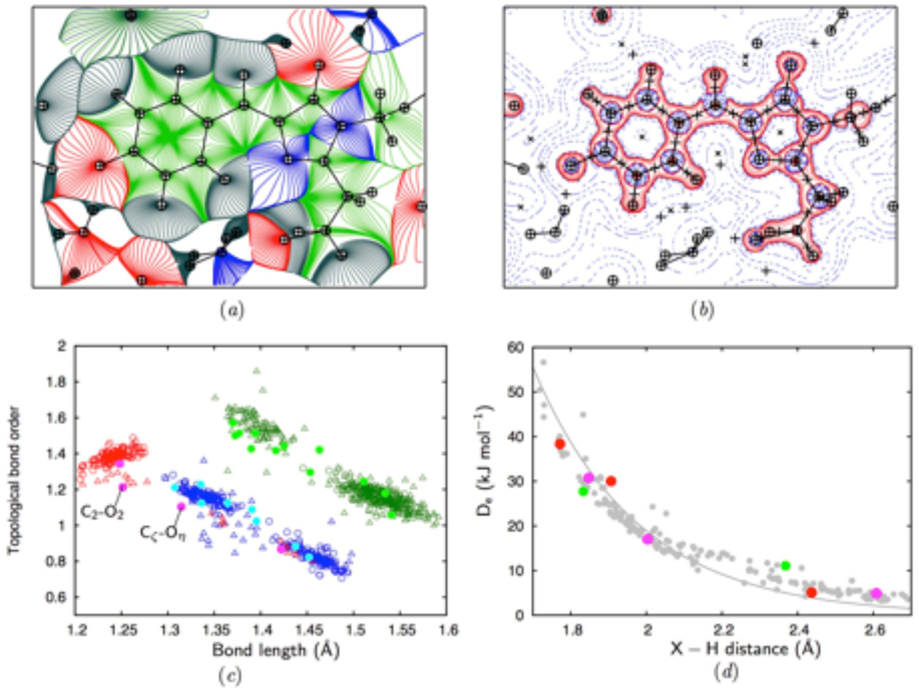

Figure S5

Topological analysis based on the electron density of MAM. (*a*) The gradient vector field around the chromophore. Atoms and vector lines are colored green for carbon, blue for nitrogen, red for oxygen and dark-green for hydrogen. (*b*) The Laplacian $\nabla^2\rho$ map around the chromophore. The view is the same as in Fig 6*a*. The contour interval is 0.05 e Å^{-5}. Red solid and blue dashed lines represent negative and positive levels, respectively. The BCPs and the ring critical points (RCP) are represented as "+" and "×", respectively. (*c*) Bond lengths and orders (Tsirelson *et al.*, 2007) for covalent bonds. Filled circles in bright-green, cyan and magenta are for C–C, C–N and C–O bonds of the chromophore, respectively. Empty circles and triangles indicate bonds of the main-chain and side-chain in a selected region for MAM. Those in green, blue and red are presented in the same manner as the chromophore bonds. (*d*) X (Acceptor)–H distances and dissociation energies for H-bonds. A relationship derived from small molecules is overlaid as a solid curve in gray (Espinosa & Molins, 2000). Filled circles in red, magenta and green are for XH---O_η, XH---O_2 and other H-bonds with chromophore, respectively. Small gray circles represent the H-bonds in the other portion of the selected region for MAM.

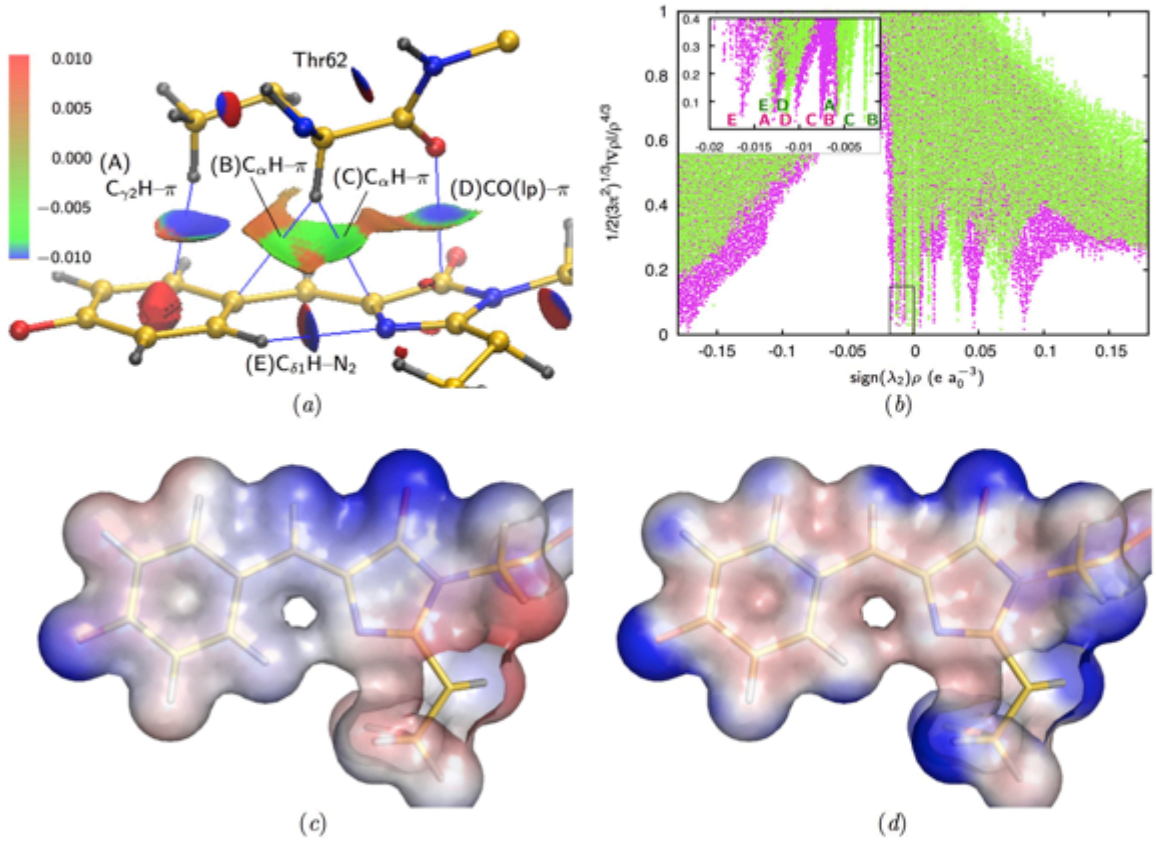

Figure S6

Non-covalent interactions on the chromophore surface. (*a*) Non-covalent interaction surfaces over the chromophore derived from the ISAM electron density. The reduced density gradient, $s(\rho)$ = 0.4 isosurface is colored according to a blue-green-red scale over the range $-0.01 < \text{sign}(\lambda_2)\rho < 0.01$ e a_0^{-3}. Blue indicates attraction, green indicates very weak attraction and red indicates repulsion. The “lp” is abbreviation of the “lone pair”. (*b*) Plots of the reduced density gradient versus the electron density multiplied by the sign of the second Hessian eigenvalue for ISAM (magenta) and MAM (green). (Inset) Details of the boxed region describing the attractive interactions. The letters, A–E, identify the corresponding interactions for each spikes. (*c*) Electrostatic potentials on the chromophore surface derived from surrounding residues of the MAM. (*d*) Electrostatic potentials on the chromophore surface derived from the ISAM. The surface is colored using a blue-white-red scale.

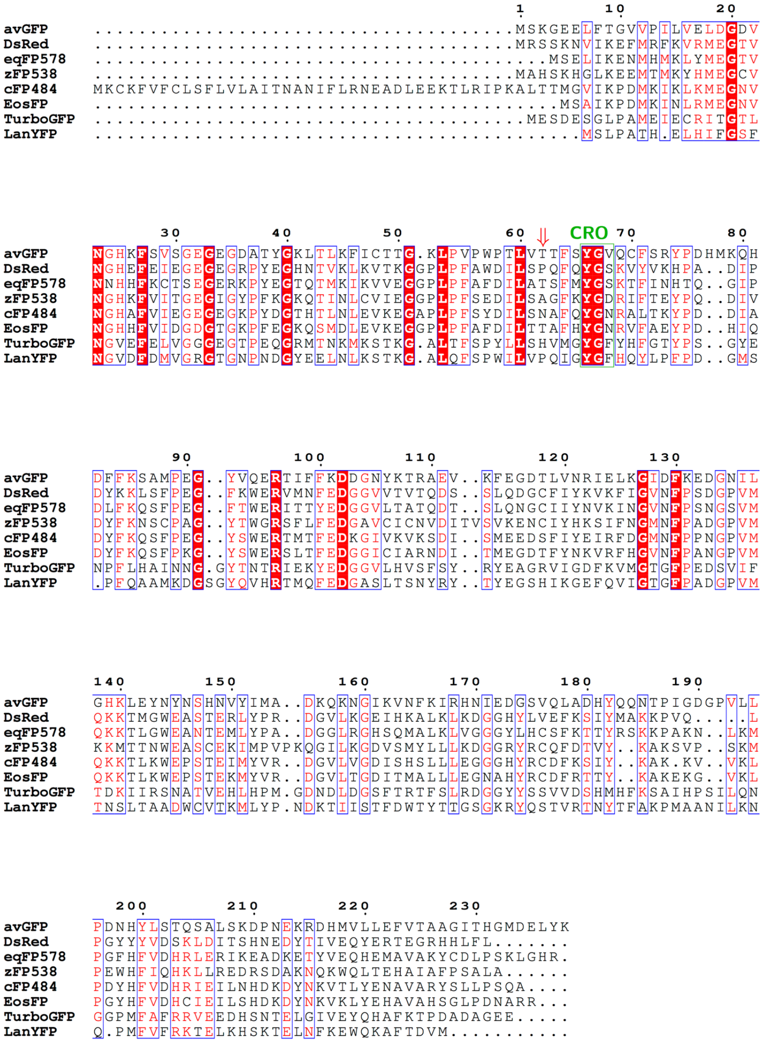

Figure S7

Sequence alignment of various colors of FPs. Strictly conserved and relatively conserved residues are shaded red and light red, respectively, with blue frames. The three continuous residues forming the chromophore are indicated with "CRO" in green letter on the sequence of ^{65}SYG^{67} (number in GFP from *Aequorea victoria*, "avGFP"). The four residues before the conserved Tyr, Thr62 in avGFP, is indicated with red arrow on the sequence.

Table S1

Data collection and crystallographic statistics for datasets for the radiation damage assessment.

Temperature (K)	Data#	Dose (MGy)	Cell dimensions *a*, *b*, *c* (Å)	$I/\sigma(I)$	R_{merge}‡ (%)	$CC_{1/2}$ (%)	Wilson *B* ($Å^2$)
50	1-1	2.0×10^4	50.88, 62.34, 69.08	0.88	95.9	55.1	11.68
	1-2	4.0×10^4	50.88, 62.34, 69.08	0.89	95.4	54.8	11.70
	1-3	6.0×10^4	50.88, 62.34, 69.08	0.90	96.9	57.4	11.70
	1-4	8.0×10^4	50.88, 62.34, 69.08	0.89	95.6	53.4	11.67
	1-5	1.0×10^5	50.88, 62.34, 69.08	0.90	95.9	54.8	11.68
	1-6	1.2×10^5	50.88, 62.34, 69.08	0.90	96.4	55.1	11.70
	1-7	1.4×10^5	50.88, 62.34, 69.08	0.87	96.8	54.7	11.68
	1-8	1.6×10^5	50.88, 62.34, 69.08	0.89	96.9	55.0	11.71
	1-9	1.8×10^5	50.88, 62.34, 69.08	0.90	96.8	53.5	11.70
	1-10	2.0×10^5	50.88, 62.34, 69.08	0.89	96.9	55.1	11.69
100	2-1	2.0×10^4	50.88, 62.34, 69.08	0.74	106.5	55.1	12.15
	2-2	4.0×10^4	50.88, 62.34, 69.08	0.73	105.5	54.8	12.06
	2-3	6.0×10^4	50.88, 62.34, 69.08	0.76	104.3	57.4	12.07
	2-4	8.0×10^4	50.88, 62.34, 69.08	0.75	104.8	53.4	12.06
	2-5	1.0×10^5	50.88, 62.34, 69.08	0.76	104.8	54.8	12.07
	2-6	1.2×10^5	50.88, 62.34, 69.08	0.74	105.8	55.1	12.08
	2-7	1.4×10^5	50.88, 62.34, 69.08	0.74	105.6	54.7	12.06
	2-8	1.6×10^5	50.88, 62.34, 69.08	0.74	106.7	55.0	12.10
	2-9	1.8×10^5	50.88, 62.34, 69.08	0.74	106.1	53.5	12.07
	2-10	2.0×10^5	50.88, 62.34, 69.08	0.73	106.1	55.1	12.09

†The highest resolution shells for all data are 1.27-1.20 Å. The $I/\sigma(I)$, R_{merge}, and $CC_{1/2}$ values are for the highest resolution shells.

‡$R_{merge} = \Sigma_{hkl}\Sigma_i| I_{hkl,i} - \langle I_{hkl}\rangle |/\Sigma_{hkl}\Sigma_i I_{hkl,i}$.

Table S2

Summary of high-resolution GFP or GFP-like crystal structures.

PDBID	Form	Resolution (Å)	Program	e.s.d (Å) †	DPI (Å) ‡	τ (°) §	φ (°) ¶	Mutation
4GF6	A	1.1	Refmac	-	0.059	-32.0	18.6	F99S/M153T/V163A
2AWK	A	1.15	Shelx	-	0.059	9.3	-7.8	S65T/F64L/R96M/F99S/M153T/V163A
3SRY	A	1.16	Phenix	-	0.056	-5.1	15.0	S65G/S72A/Q183A/T203Y/H231L
3ST0	A	1.19	Phenix	-	0.066	9.0	-2.1	S65G/Q69T/S72A/K79R/V163A/T203Y /H231L
2QZ0	A	1.2	Shelx	-	0.078	-10.1	11.0	F64L/F99S/M153T/V163A/Q183E
4GES	A	1.23	Refmac	-	0.079	-42.5	25.2	F99S/M153T/V163A
3DQ7	A	1.23	Refmac	-	0.138	0.5	8.1	S65G/V68L/Q69M/S72A/T203Y/H231L
3SSH	A	1.28	Phenix	-	0.070	-2.5	12.4	S65G/S72A/K79R/Q183A/T203Y/H231L
3GJ1	A	1.8	Refmac	-	0.235	7.4	2.8	F99S/M153T/V163A/T203H
2WUR	ambiguous	0.9	Shelx	-	0.034	-1.0	-1.3	F64L/I167T/K238N
2FZU	ambiguous	1.25	Shelx	-	0.072	1.1	7.4	S65T/F64L/F99S/M153T/V163A
1GFL	ambiguous	1.9	X-plor	-	0.300	0.5	0.0	-
2HQZ	B	1.2	Shelx	-	0.057	7.4	-7.1	S65T/L42H/F64L
2DUH	B	1.2	Shelx	-	0.080	4.2	-4.4	S65T/H148D
2DUE	B	1.24	Shelx	-	0.075	5.7	-4.2	S65T/H148N
2HJO	B	1.25	Shelx	-	0.065	8.5	-7.2	S65T/F64L/V224H
1EMA	B	1.9	TNT	-	0.328	17.6	-13.0	S65T
1EMG	B	2.0	TNT	-	0.298	4.0	-4.2	S65T
6JGH (T203I)	A	0.94	Shelx	0.012	0.023	0.6	-5.6	F99S/M153T/V163A/T203I
6JGI (S65T)	B	0.85	Shelx	0.007	0.016	-0.5	-1.9	S65T/F99S/M153T/V163A
6JGJ (E222Q)	B	0.78	Shelx/MoPro	0.006/0.007	0.015/0.014	-1.0	-0.4	F99S/M153T/V163A/E222Q

†Estimated standard deviations for bond lengths derived from the full-matrix refinement with *SHELXL* or *MoPro*. The average values for the chromophore.

‡Dispersion Precision Indicator as positional error calculated with the data deposited in PDB as follows: $\sigma(r, B_{avg}) = 3^{1/2}(N_i/p)^{1/2}C^{-1/3}Rd_{min}$ (Cruickshank, 1999).

§A dihedral angle (tilt) defined with N_2–$C_{\alpha 2}$–$C_{\beta 2}$–C_{γ}.

¶A dihedral angle (twist) defined with $C_{\alpha 2}$–$C_{\beta 2}$–C_{γ}–$C_{\varepsilon 1}$.

Table S3

Conditions in theoretical calculations on chromophore geometry.

	Ref.†	Ini‡	Residues in QM calculation							
			Arg96§	His148	Thr203	Ser205	Glu222	Wat3	Thr62	Others‖
**With protein environment¶**										
Sinicropi et al., 2005	1	1GFL	-	-	-	-	-	○	-	2 waters
Altoè et al., 2007	2	1GFL	-	-	-	-	-	○	-	2 waters
Bravaya et al, 2011	3	1EMA	○	○	-	○	○	○	-	1 water
Amat & Nifosì, 2013	4	1EMG	○	○	○	○	○	○	○	Tyr145, Gln69, Gln94, 3 waters
Grigorenko et al, 2013	5	1EMA	-	○	○	○	○	○	-	-
Ding et al., 2013	6	3GJ1	-	-	-	-	○	-	-	-
Beerepoot et al., 2013	7	1GFL	-	-	-	-	-	-	-	-
**Isolated¶**										
Tozzini & Nifosì, 2001	8	-	-	-	-	-	-	-	-	-
Martin et al., 2004	9	-	-	-	-	-	-	-	-	-
Altoe et al., 2005	10	-	-	-	-	-	-	-	-	-
Olsen & Smith, 2008	11	-	-	-	-	-	-	-	-	-
Filippi et al., 2009	12	-	-	-	-	-	-	-	-	-
Amat & Nifosì, 2013	4	-	-	-	-	-	-	-	-	-
Beerepoot et al., 2013	7	-	-	-	-	-	-	-	-	-

†Reference numbers correspond with the number in Fig 4.

‡A PDB-ID for the initial model structure in the optimization. Also refer Table S2.

§Representative residues considered in the QM region of the calculations are marked with "○", while the unconsidered are marked with "-".

‖Uncommon residues in each calculation.

¶The calculations with and without surrounding residues are classified as "With protein environment" and "Isolated", respectively. The majority of the protein residues were treated in MD, excluding those listed in the table.

Table S4

H-bond distances around the chromophore.

H-Donor	Acceptor	T203I d_{D-A} (Å)	S65T d_{D-A} (Å)	E222Q d_{D-A} (Å)
N-Arg168	$N_{\varepsilon2}$-His148	3.050(70)‡	3.157(9)	3.150(30)‡
$N_{\delta1}$-His148	O_{η}-Tyr66	-	2.869(8)	2.867(7)
O_{η}-Tyr66	Wat3	2.623(11)	-	-
Wat3	O_{η}-Tyr66	-	2.737(7)	2.727(7)
Wat3	O-Asn146	2.872(11)	2.903(7)	2.860(7)
Wat3	O_{γ}-Ser205	2.275(11)	-	-
O_{γ}-Ser205	Wat3	-	2.762(7)	2.698(6)
O_{γ}-Ser205	$O_{\varepsilon2}$-Glu222	2.609(13)‡	-	2.799(8)‡
$O_{\gamma1}$-Ser65	$O_{\varepsilon1}$-Glu222 ($O_{\varepsilon1}$-Gln222)	2.661(12)‡	-	2.742(7)‡
$O_{\varepsilon1}$-Glu222 ($N_{\varepsilon1}$-Gln222)	$O_{\gamma1}$-Ser65	-	2.670(6)‡	-
$O_{\gamma1}$-Ser65	N_2-Tyr66	-	2.740(7)	-
O_{γ}-Thr203	O_{η}-Tyr66	-	2.672(6)	-

† Values in parentheses are estimated standard deviations derived from the full-matrix least squares refinement.

‡ There values are for the major of the alternative conformations.

Table S5

Properties of H-bonding† around the chromophore.

H-Donor	Acceptor	$d_{D\text{-}A}$ (Å)	$d_{H\text{-}A}$ (Å)	ρ_{BCP} (e Å^{-3})	$\nabla^2\rho_{BCP}$ (e Å^{-3})	D_e (kJ mol^{-1})
Wat3	O_η-Tyr66	2.727(7)	1.77(10)	0.188	4.20	38.8
$N_{\delta1}$-His148	O_η-Tyr66	2.867(7)	1.91(6)	0.180	2.64	30.1
$C_{\varepsilon2}$-Tyr145	O_η-Tyr66	3.517(9)	2.44(8)	0.026	0.97	5.1
$N_{\eta2}$-Arg96	O_2-Tyr66	2.760(6)	1.85(8)	0.168	3.27	30.8
$N_{\varepsilon2}$-Gln94	O_2-Tyr66	2.940(7)	2.00(9)	0.094	2.40	17.0
C_γ-Gln69	O_2-Tyr66	3.369(7)	2.61(7)	0.046	0.68	4.9
$O_{\gamma1}$-Ser65	$O_{\varepsilon1}$-Gln222	2.742(7)	1.83(8)	0.160	2.86	27.8
$C_{\delta1}$-Tyr66	N_2-Tyr66	3.113(7)	2.37(6)	0.089	1.22	11.1

†The bonding is defined based on AIM theory. A critical point where $\nabla\rho_{BCP}=0$ and $\rho_{BCP}>0$ should be detected between a donor and an acceptor.

‡ Values in parentheses are estimated standard deviations derived from the full-matrix least squares refinement.
